# Principles and Practice of Obstetric Medicine and Surgery, in Reference to the Process of Parturition

**Published:** 1855-03

**Authors:** 


*The Principles and Practice of Obstetric Medicine and Surgery, in reference to the Process of Parturition.* With sixty-four plates and numerous wood cuts. By FRANCIS H. RAMSBOTHAM, M. D. A new American edition, revised by the author. With notes and additions, by WILLIAM Y. KEATING, M. D., A. M., Lecturer on Obstetrics and Diseases of women in the Philadelphia Medical Association, etc., etc. Philadelphia: Lea & Blanchard. 1855. 8vo. pp. 648.

---

THIS, the third edition of the great work of Dr RAMSBOTHAM, has been rendered more useful than either of the former, as well as beautified, by an additional number of illustrations, well calculated to explain and illustrate the text.

We have already recommended this work to every student who desires to acquire knowledge, and to every practitioner who wishes to refresh his memory, as a most faithful picture of practical midwifery; and we can in all candor say, that altogether it is one of the best books we have seen on the subject of which it treats. The style is clear, concise and simple—the illustrations embrace every essential point of practical importance and are in the first style of excellence. The additions of the American editor are calculated to adapt the work to the wants of practitioners in this country, and, although we might as a western man, be disposed to inquire whether the two Philadelphia professors are the only obstetric authorities in the United States, still we are willing to receive the volume as a satisfactory exhibition of both English and American Midwifery, and to commend it with great cordiality to our readers.

The present edition contains a number of valuable additions both by the author and editor.

The publishers, Messrs. LEA & BLANCHARD, have done themselves great credit by the elegance with which they have executed their portion of the work, having succeeded in furnishing one of the most beautiful volumes ever seen in the physician's library.

